# *Leishmania major* large RAB GTPase is highly immunogenic in individuals immune to cutaneous and visceral leishmaniasis

**DOI:** 10.1186/s13071-017-2127-3

**Published:** 2017-04-17

**Authors:** Rym Chamakh-Ayari, Mehdi Chenik, Ahmed Sahbi Chakroun, Narges Bahi-Jaber, Karim Aoun, Amel Meddeb-Garnaoui

**Affiliations:** 1 Laboratory of Medical Parasitology, Biotechnology and Biomolecules, LR11-IPT-06, Institut Pasteur de Tunis, Tunis, Tunisia; 2 University of Carthage, Tunis, Tunisia; 3 Molecular Epidemiology and Experimental Pathology Applied to Infectious Diseases Laboratory, Institut Pasteur de Tunis, Tunis, Tunisia; 4 Institut Polytechnique LaSalle Beauvais, Beauvais, France

**Keywords:** RAB GTPase, Vaccine, Human leishmaniasis, *Leishmania*, Cross-protection

## Abstract

**Background:**

We previously identified a *Leishmania* (*L.*) *major* large RAB GTPase (LmlRAB), a new atypical RAB GTPase protein. It is highly conserved in *Leishmania* species while displaying low level of homology with mammalian homologues. *Leishmania* small RAB GTPases proteins have been involved in regulation of exocytic and endocytic pathways whereas the role of large RAB GTPases proteins has not been characterized yet. We report here the immunogenicity of both recombinant rLmlRAB and rLmlRABC, in individuals with immunity against *L. major* or *L. infantum*.

**Methods:**

PBMC were isolated from individuals cured of *L. major* (CCLm) or from healthy individuals. The latter were subdivided into high or low IFN-γ responders. Healthy high IFN-γ responders, considered as asymptomatics, were living in an endemic area for *L. major* (HHR*Lm*) or *L. infantum* (HHR*Li*). Healthy low IFN-γ responders (HLR) were considered as naïve controls. Cells from all volunteers were stimulated with rLmlRAB or rLmlRABC. Cytokines were analysed by CBA and ELISA and phenotypes of IFN-γ-producing cells were analysed by flow cytometry.

**Results:**

Both rLmlRAB and rLmlRABC induced high significant levels of IFN-γ in CCLm, HHR*Lm* and HHR*Li* groups. Phenotype analysis of rLmlRAB and rLmlRABC-stimulated T cells in CCLm individuals showed a significant increase in the percentage of specific IFN-γ-producing CD4+ and CD8+ T cells. rLmlRAB induced significant granzyme B levels in CCLm and HHR*Lm*. Low but significant granzyme B levels were detected in naïve group. IL-10 was detected in immune and naïve individuals.

**Conclusion:**

We showed that rLmlRAB protein and its divergent carboxy-terminal part induced a predominant Th1 response in individuals immune to *L. major* or *L. infantum*. Our results suggest that rLmlRAB and rLmlRABC proteins are potential cross-species vaccine candidates against cutaneous and visceral leishmaniasis.

## Background

Leishmaniasis is a vector-borne disease caused by intracellular protozoan parasites belonging to the genus *Leishmania*. This disease is characterized by a spectrum of clinical manifestations determined by *Leishmania* species and the host immune response against the parasite. It ranges from asymptomatic infections to cutaneous or fatal visceral forms [1]. The control of leishmaniasis mainly relies on chemotherapy, which has been hampered by drug toxicity and the emergence of drug resistant strains [2]. In endemic areas, healing appears to confer life-long immunity to re-infection suggesting the feasibility of a vaccine in humans. Cell-mediated immunity is essential for resistance and the establishment of a protective immunity against infection. The cellular immune responses developed during leishmaniasis have been extensively studied in mouse models, mainly using *Leishmania* (*L.*) *major* infection. In resistant mice, T-helper 1 (Th1)-mediated response promotes interferon-γ (IFN-γ) production, which activates infected macrophages to kill parasites *via* nitric oxide production leading to the control of parasite burden and lesion healing [3, 4]. Resistance to re-infection is mediated by the generation of memory CD4+ T cells in these mice [5]. Conversely, in susceptible BALB/c mice, a Th2 dominant response is observed, leading to the production of anti-inflammatory cytokines, such as IL-4, IL-5 and IL-13, which promotes disease progression in human leishmaniasis, a clear Th1- or Th2-polarized immune response similar to murine model is never observed. However, healing and resistance to re-infection is generally correlated with the development of a dominant antigen-specific Th1 cell responses and IFN-γ production [5]. CD4+ T cells as well as CD8+ cells play major roles in the healing process during human *Leishmania* infection, mainly through IFN-γ production [6–8]. More recently, a balance between the proportion of CD4+ and CD8+ cells has been reported to be important for leishmaniasis healing. Multifunctional T CD4+ cells producing IFN-γ, TNF-α and IL-2 were correlated with protection against murine experimental leishmaniasis and were recently detected in individuals healed of *L. braziliensis* infection. Many studies have focused on the identification of antigens inducing *Leishmania*-specific CD4+ and CD8+ T cell responses [9, 10]. Several *Leishmania* recombinant proteins have been investigated as vaccine candidates in animal models and variable results regarding the ability to induce protection were observed. A number of *Leishmania* proteins were specifically recognized by Th1 cells of humans exposed to the parasite and were considered as potential vaccine candidates [9–12]. However, to date there is yet no protective vaccine against human leishmaniasis.

We previously identified a new atypical *L. major* large RAB protein, LmlRAB [13]. LmlRAB (610 aa) displays a conserved domain located in amino-terminal part (34–284) and contains characteristic signatures of RAB proteins, GTP binding domains, RAB specific domains, RAB subfamily-specific domains and a prenylation site in the last 4 amino acids. In addition, LmlRAB shows a divergent carboxy-terminal domain (LmlRABC) (285–610) that is specific to *Leishmania* species [13]. RAB GTPases are well known for their key role in regulation of exocytic and endocytic pathways in eukaryotic cells and more globally as specialized trafficking pathways proteins that are involved in the fusion of phagosomes with various endocytic organelles [14]. Several Rab GTPases genes were identified in *Leishmania* parasites genomes *in silico*. However, only some of them have experimentally been shown to play a role in the regulation of exocytic and endocytic pathways. Rab5 plays a major role in hemoglobin trafficking and Rab7 is involved in the degradation of endocytosed hemoglobin, which is required for optimal *Leishmania* growth [15, 16]. Recently, Bahl et al. [17] also showed that Rab1 plays an essential role in the regulation of the secretory pathway in *Leishmania.* An isoform of Rab5 was described as a specific regulator of different modes of endocytosis [18].

To identify vaccine candidate molecules against human leishmaniasis, we evaluated the ability of the recombinant LmlRAB (rLmlRAB) and its divergent carboxy-terminal part, rLmlRABC, to induce cellular immune responses in individuals with protective immunity against *L. major* or *L. infantum* infection.

## Methods

### Study population and samples

Peripheral blood samples were collected from 91 donors (age ranging from 18 to 60 years). Human groups included individuals who have recovered from CL due to *L. major* (CCLm) and healthy individuals with no history of leishmaniasis but with a probable asymptomatic infection. These individuals were recruited from two well-characterized endemic foci located in central Tunisia for cutaneous leishmaniasis (CL) due to *L. major* (Guettitir) and visceral leishmaniasis (VL) due to *L. infantum* (Dkhila).

Cured individuals were recruited based on the following criteria: (i) living in endemic *L. major* foci; (ii) well-documented medical records; (iii) presence of typical scars; and (iv) high IFN-γ response to Soluble *Leishmania* Antigens (SLA) (>300 pg/ml). Healthy individuals with a probable asymptomatic *L. major* (Healthy High Responders, HHR*Lm*) or *L. infantum* infection (HHR*Li*), were recruited from an endemic area for CL or VL, respectively, with high IFN-γ response to SLA (> 300 pg/ml) and no scars. Healthy individuals with no or low IFN-γ response to SLA (< 100 pg/ml) (Healthy Low Responders, HLR) and with no history of leishmaniasis, were recruited from Tunis, a low endemicity area, and were considered as naïve controls. Exclusion criteria were immunosuppressive diseases other than leishmaniasis, long-term treatment and pregnancy. The different human groups used in this study are detailed in Table 1.

**Table 1:** Study population

Status	Group ID	Number	Average age	*Leishmania* species
Cured from CL	CCLm	30	43.34 ± 13.07	*L. major*
Healthy high responder (*L. major*)	HHR*Lm*	18	39.17 ± 10.40	*L. major*
Healthy high responder (*L. infantum*)	HHR*Li*	26	35.75 ± 9.55	*L. infantum*
Healthy low responders	HLR	17	30.76 ± 5.73	–

*Abbreviations*: *CCLm* Cured from cutaneous leishmaniasis due to *L. major*, *HHRLm* Healthy High Responders (*L. major*), *HHRLi* Healthy High Responders (*L. infantum*), HLR, naïves control

The recruitment and peripheral blood sampling of different groups including cured from CL due to *L. major* (CCLm) and individuals with a probable asymptomatic *L. major* (HHR*Lm*) or *L. infantum* infection (HHR*Li*), were performed in endemic foci for CL due to *L. major* and VL due to *L. infantum*. Healthy individuals with no history of leishmaniasis, used as naïve controls (HLR) were recruited from a low-endemic area

### Cloning of LmlRAB and plasmid constructions

The full-length Lmlrab gene (1,833 bp; GenBank: AY962589) and LmlrabC, corresponding to the divergent carboxy-terminal part (1,286 bp) were amplified by PCR from genomic DNA of *L. major* (MHOM/TN/94/GLC94) using specific primers (Table 2). The purified PCR products were digested with specific restriction enzymes and cloned in pET-22b (+) expression vector (Novagen, EMD Millipore). The transformed BL21 *E. coli* were screened for the presence of recombinant plasmid with the Lmlrab or LmlrabC insert by gene-specific PCR. Isolated positive clones were subsequently sequenced.

**Table 2:** Primers used for cloning of LmlRAB and LmlRABC and plasmid constructions

Name	Type	Primer sequences	Primer sequences	Restriction site
F-Ras1a	Forward	rlmlrab	5′-GCCCATATGAGCTCAACTGGTCAGCATG-3′	NdeI
F-Ras3a	Forward	rlmlrabC	5′GCCCATATGATCATCGCCGATGTGTCG-3′	NdeI
R-Ras3b	Reverse	rlmlrab/rlmlrabC	5′-GCCGCGGCCGCCCAGTACGCAGCAGTTGC-3′	NotI

Reverse and forward primers containing at their 5′ end a restriction site (underlined) were used to amplify the full length LmlRAB ORF and its carboxyl part LmlRABC. The amplified double-stranded DNA were first digested with NdeI and NotI and then inserted into the corresponding cloning sites in the pET-22b + vector (Novagen)

### Production of purified recombinant rLmlRAB and its divergent carboxy-terminal part rLmlRABC

**Fig. 1:**
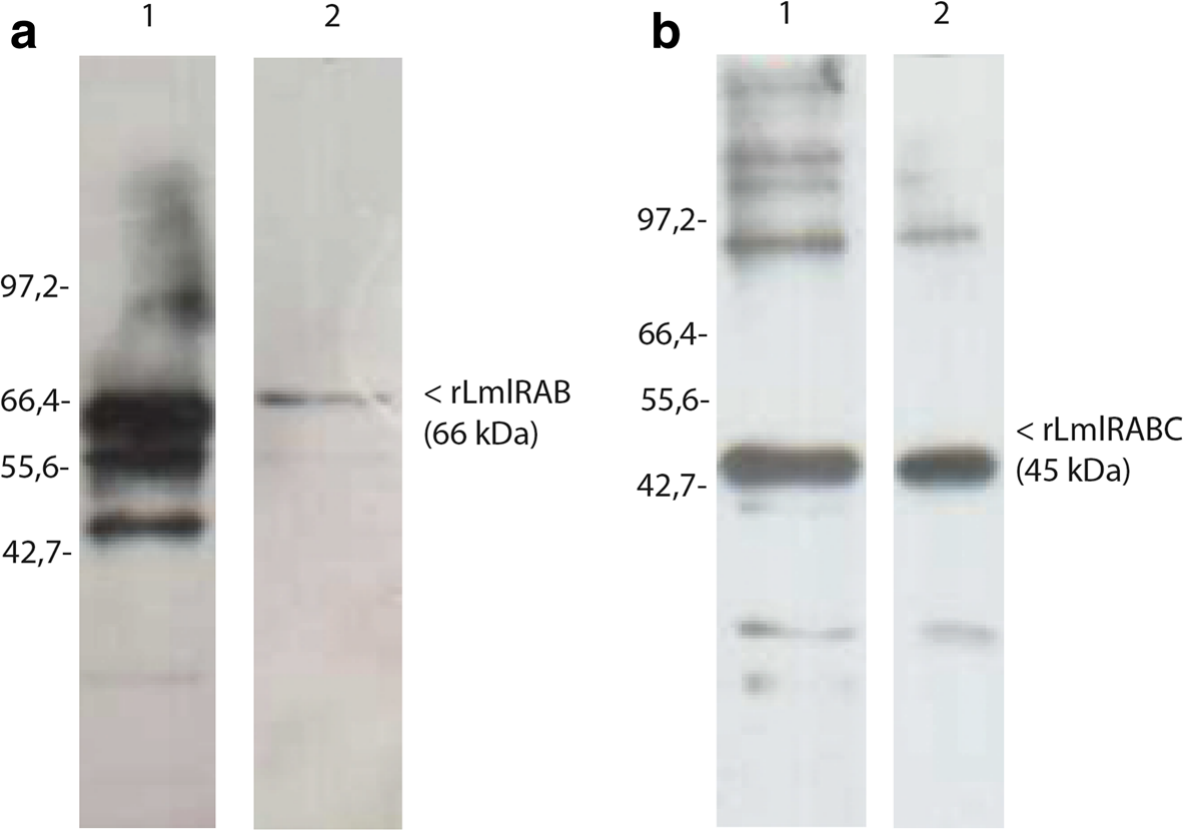
Expression of recombinant rLmlRAB and rLmlRABC in *E. coli*. Recombinant rLmlRAB as well as its carboxyl terminal part rLmlRABC were synthesized in BL21, purified by affinity chromatography over Ni-NTA resin. Western blot analysis shows the reactivity of the polyclonal anti-LmlRAB against the insoluble fraction of *E. coli* expressing both proteins rLmlRAB (**a** Lane 1) and LmlRABC (**b** Lane 1). The purified proteins rLmlRAB (**a** Lane 2) and rLmlRAB (**b** Lane 2) were also analyzed

BL21 *E. coli* strain cells harboring the recombinant plasmid pET-LmlRAB and pET-LmlRABC were grown in LB medium, induced with 1 mM isopropyl-1-thio-β-d-galactopyranoside (IPTG) for 4 h and lysed. Recombinant LmlRAB-(His)_6_ (rLmlRAB) and LmlRABC-(His)_6_ (rLmlRABC) were synthesized as insoluble proteins. These proteins were solubilized in 6 M guanidine-HCl and purified by affinity chromatography over Ni-NTA resin using an imidazole gradient elution (GE Healthcare, Biosciences, Uppsala, Sweden). The Purity was demonstrated by 12% SDS-polyacrylamide gel followed by Coomassie blue staining (data not shown). Western blot analysis was performed using polyclonal antibodies directed against the divergent carboxy-terminal part rLmlRABC (Fig. 1).

Purified recombinant proteins were also tested for the presence of LPS using 10 μg/ml of polymyxin B (Sigma-Aldrich, Steinheim, Germany) or 100 μg/ml of proteinase K (Invitrogen, Darmstadt, Germany) in PBMCs stimulated cultures. IL-10 production was strongly inhibited by proteinase K treatment whereas polymixin B did not affect this activity, indicating the absence of LPS contamination in the purified recombinant proteins.

### Analysis and comparison of DNA and amino acid sequences

Nucleotide and deduced amino acid sequences of LmlRAB were downloaded from the GenBank database using the ID: AY962589.1 and used as a query to run the NCBI-Blast program hosted on the online Kinetoplatid Genomics Ressources (tritrypdb.org). The similarity analysis of LmlRAB sequences was performed over the whole genome sequences of the following *Leishmania* strains (*L. major* strain Friedlin; *L. major* strain SD 75.1; *L. major* strain LV39c5; *L. infantum* strain JPCM5; *L. donovani* strain BPK282A1; *L. tropica* strain L590; *L. mexicana* strain U1103; *L. amazonensis* strain M2269; *L. aethiopica* strain L147; *L. braziliensis* strain M2904 and *L. braziliensis* strain M2903). The corresponding genomic sequences were downloaded from the TriTrypDB plateform and translated into amino acid sequences using Geneious Pro version (3.6.2) [19]. Pairwise and overall global alignments were generated. Finally, the domains annotations were edited over the alignment to assess global and local similarities.

### Preparation of soluble *Leishmania* promastigote antigens (SLA)

SLA were prepared from promastigote stationary phase parasite cultures of *L. major* (MHOM/TN/94/GLC94). Parasites were washed in 1× phosphate-buffered saline (PBS), centrifuged at 1,000× g/10 min at 4 °C and supernatants were removed. The pellets were resuspended in lysis buffer (50 mM Tris/5 mM EDTA/HCl, pH7.1 ml/1 × 10^9^ parasites), subjected to three rapid freeze/thaw cycles followed by three pulses of 20 s/40 W with sonicator. Samples were centrifuged at 5,000× *g* for 20 min at 4 °C, and supernatants were collected, aliquoted and stored at -80 °C until use. Protein quantification was performed using Bradford method.

### Cell culture and stimulation

Peripheral blood mononuclear cells (PBMCs) were isolated from blood by density centrifugation through Ficoll-Hypaque (GE Healthcare Bio-Sciences AB, Uppsala, Sweden). Cells were cultured in RPMI 1640 supplemented with 10% heat inactivated FBS, 100 IU/ml penicillin, 100 μg/ml streptomycin, 2 mM L-glutamin, 50 μM 2-mercaptoethanol, 1 mM sodium pyruvate and 1× non essential amino acid. Briefly, cells were plated in 96 well tissue culture plates (TPP, Switzerland) and were kept with media alone (unstimulated) or stimulated with 10 μg/ml phytohemagglutinin (PHA) (Sigma-Aldrich) used as a positive control or 10 μg/ml SLA or 10 μg/ml rLmlRAB or rLmlRABC, in a 5% CO_2_ humidified atmosphere at 37 °C for 5 days.

### ELISA

IFN-γ and Interleukine-10 (IL-10) levels were measured, by Enzyme-linked immunosorbent assay (ELISA), in cell culture supernatants that were collected after 120 h, centrifuged and stored at -80 °C until use. Human IFN-γ or IL-10 ELISA Sets (BD Biosciences) were used according to manufacturer’s instructions. Results were interpolated from a standard curve using recombinant cytokines and were expressed in pg/ml.

### Cytometric Bead Array assay (CBA)

Granzyme B and tumor necrosis factor-alpha (TNF-α) were detected and quantified from culture supernatants (50 μl) of PBMC exposed for 120 h to PHA (10 μg/ml), SLA (10 μg/ml), rLmlRAB or rLmlRABC (10 μg/ml), using the BD CBA Human Soluble Protein Flex Set system, according to the instructions of the manufacturer (BD Biosciences). Granzyme B was analysed only in CCLm, HHR*Lm* and HLR groups. In order to quantitate samples, the BD™ CBA Human Soluble Protein Flex Standard was performed for each cytokine and in each experiment. Data were acquired by flow cytometry (FACS Canto II, BD Bioscience) using 2-color detection. Flow Cytometric Analysis Program Array (FCAP Array™; BD Biosciences) software was used for samples analysis.

### Intracellular cytokine staining and flow cytometry

Freshly isolated PBMC from CCLm group were stimulated with PMA (50 ng/ml)/ionomicyn (10^−6^M) for 6 h (positive control) or SLA (10 μg/ml) or rLmlRAB or rLmlRABC (10 μg/ml) for 120 h, or kept with medium alone. Cells were treated with Golgistop (BD Biosciences) for the last 6 h of culture, then washed and incubated with antibodies: FITC CD3, PerCPcy5.5 CD4, APC-H7 CD8 or PerCPcy5.5 CD8, and PE-Cy7 CD69 or PE CD69 (BD Biosciences), for 20 min at 4 °C. For intracellular IFN-γ detection, cells were fixed and permeabilized using BD Cytoperm/cytofix plus kit (BD Biosciences) according to manufacturer’s instructions and labelled with PE-anti-IFN-γ mAb (intracellular formulation) (BD Biosciences). BD™ CompBeads Set Anti-Mouse Ig, κ (Anti-Mouse Ig, κ/Negative Control (FBS) Compensation Particles Set) (BD Biosciences) was used for compensation controls. Analysis was performed with DIVA software.

### Statistical analysis

Data analysis was performed with Stata statistical software (StataCorp. 2009. Stata Statistical Software: Release 11. College Station, TX: StataCorp LP.). We used non-parametric statistical tests because of the low number of individuals in some groups and of the heterogeneity of the standard deviation between groups. Results are expressed as mean ± standard deviation (SD) and a *P*-value of < 0.05 was considered significant in all cases (granzyme B and TNF-α). In the statistical analysis of cytokine level and phenotyping, we used Wilcoxon signed-rank test to compare median levels of cytokines (IFN-γ and IL-10) or percentage of cells producing IFN-γ after different stimulations in paired data. Kruskal-Wallis rank test was used for inter-groups analysis on normalized data after deducting the non-stimulated value.

## Results

### LmlRAB is highly conserved among *Leishmania* species

Screening of effective *Leishmania* vaccine candidates requires the identification of antigens that are highly conserved among *Leishmania* species. We previously showed that the LmlRAB is highly conserved among *Leishmania* strains and species [13]. In order to confirm this finding and given the availability of recent data on *Leishmania* genome, we performed a similarity analysis using additional *Leishmania* strains and species.

**Fig. 2:**
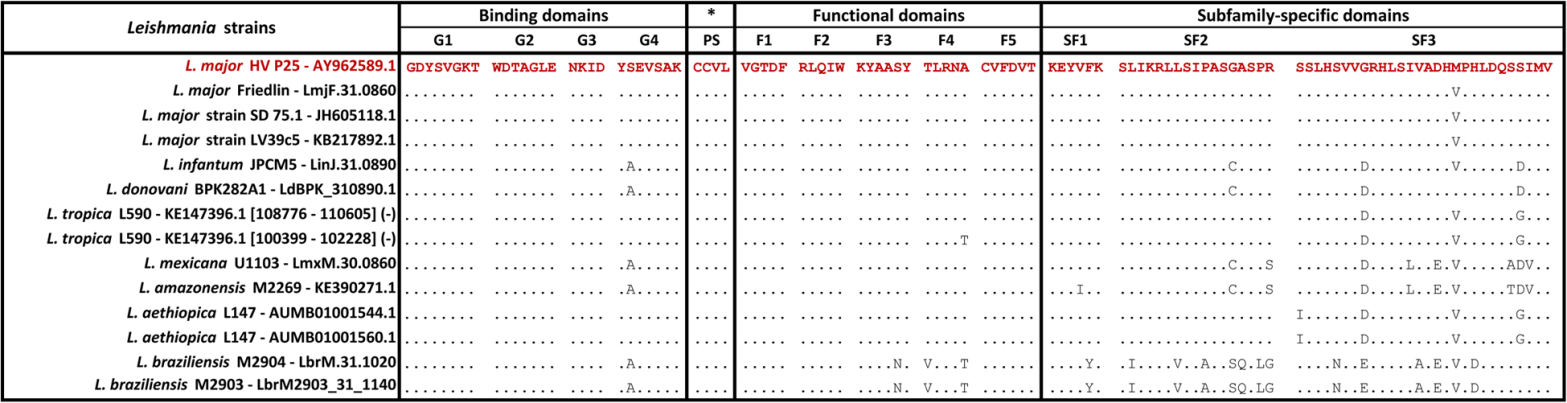
Local alignment of specific Rab GTPase like protein domains. Domains were first identified and extracted from the *L. major* HV P25 Rab GTPase like sequence and from its selection of orthologs, then aligned. The alignment was edited in a way to shed light on the variable sites by marking all identical residues with dots

As expected, *Leishmania* large RAB GTPases proteins are conserved among *Leishmania* strains and species, with similarity ratios ranging between 98.9 to 63.7% (Table 3). In addition, alignments of specific LmlRAB regions ranging from amino acid 1 to 183 and 184 to 611, respectively identified as N- and C-terminal parts, revealed that the N-terminal regions are more conserved than the C-terminal ones, with a similarity ratio span of 98.9 to 74.3% for the N-terminal and of 98.9% falling to 59.3% for the C-terminal (Table 3). Interestingly, RAB GTPases domains (binding and functional domains and the prenylation site) are highly conserved among all studied *Leishmania* strains and species (Fig. 2).

**Table 3:** Similarity ratios produced by pairwise alignment of LmlRab with *Leishmania* strains ortholog sequences

*Leishmania* strain	Chromosome/Gene ID	Genomic coordinates	Product^a^	Similarity %		Similarity %		Similarity %	
				*L. major* HV P25 (Tunisia)		N-ter		C-Ter	
				AY962589.1		AY962589.1		AY962589.1	
				N (%)	P (%)	N (%)	P (%)	N (%)	P (%)
*L. major* Friedlin	LmjF.31.0860	315900–317732 (–)	ras-like small GTPases, putative	99.60	98.90	99.5	98.9	99.6	98.8
*L. major* strain SD 75.1	JH605118.1	298109–299941 (–)	not yet annotated	99.60	98.90	99.5	98.8	99.5	98.9
*L. major* strain LV39c5	KB217892.1	1129713–1131545	not yet annotated	99.50	98.70	99.5	98.9	99.6	98.6
*L. infantum* JPCM5	LinJ.31.0890	322703–324532 (–)	ras-like small GTPases, putative	94.40	91.00	94.00	94.5	93.0	89.3
*L. donovani* BPK282A1	LdBPK_310890.1	329426–331255 (–)	not yet annotated	93.30	90.80	94.2	95.1	92.6	88.6
*L. tropica* L590	KE147396.1	108776–110605 (–)	not yet annotated	94.30	90.70	90.4	94.0	87.1	89.0
*L. tropica* L590	KE147396.1	100399–102228 (–)	not yet annotated	94.30	90.70	95.4	95.1	93.6	89.3
*L. mexicana* U1103	LmxM.30.0860	324259–326091 (–)	ras-like small GTPases, putative	93.10	90.50	96.2	90.2	88.0	79.4
*L. amazonensis* M2269	KE390271.1	700–2532	not yet annotated	94.20	90.50	90.4	89.6	87.1	78.3
*L. aethiopica* L147	AUMB01001544.1	10483–12315	not yet annotated	88.70	82.70	95.4	93.4	93.8	89.5
*L. aethiopica* L147	AUMB01001560.1	101–1933	not yet annotated	88.10	81.70	95.4	93.4	93.8	89.5
*L. braziliensis* M2904	LbrM.31.1020	360616–362445 (–)	ras-like small GTPases, putative	74.50	63.80	78.1	74.3	72.9	59.3
*L. braziliensis* M2903	LbrM2903_31_1140	408882–410711 (–)	ras-like small GTPases, putative	74.40	63.70	78.3	74.3	72.9	59.3

^a^According to TriTrypDB release 24 (15/04/2015) www.tritrypdb.org

For each of the selected *Leishmania* strains, the chromosome (or gene ID if chromosome were not assembled yet), the genomic coordinates and the gene product as described in the TriTrypDB server (Release 24 of 15/04/2015) is provided. Similarity ratios were drawn from pairwise alignments of both nucleic (columns noted “N”) and peptidic (columns noted “P”) sequences, regarding the entire gene sequence (column named “*L. major* HV P25 (Tunisia)”), the so called N-terminal region ranging from residue 1 to 183 (column named “N Ter”) and the so called C-terminal region ranging from residue 184 to 611 (column named “C Ter”)

### rLmlRAB and rLmlRABC induce a dominant Th1 cytokine pattern in individuals immune to *L. major* or *L. infantum* infection

**Fig. 3:**
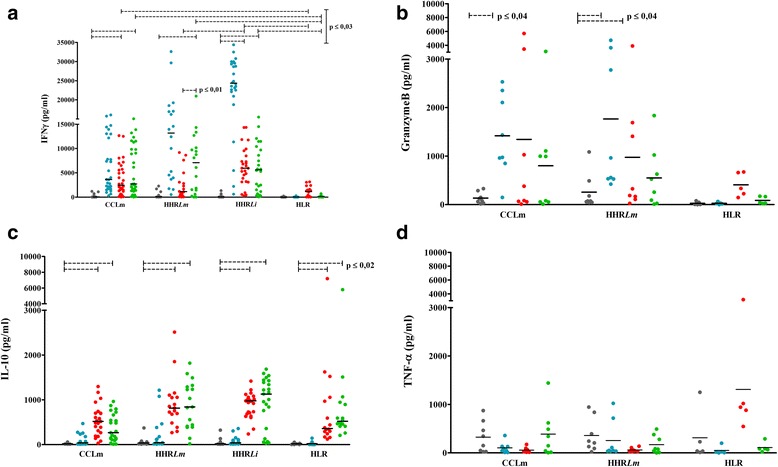
rLmlRAB and rLmlRABC specific IFN-γ, granzyme B, IL-10 and TNF-α responses. **a** IFN-γ, **b** granzyme B, **c** IL-10 and **d** TNF-α were detected and quantified from culture supernatants of PBMC (106 cells/ml) stimulated for 120 h with SLA (10 μg/ml), rLmlRAB or rLmlRABC (10 μg/ml). IFN-γ and IL-10 production were measured by ELISA. Granzyme B and TNF-α were measured using CBA and data were analyzed by flow cytometry. PHA (10 μg/ml) was used as positive control (data not shown). Bars indicate the median value for IFN-γ (**a**) and IL-10 (**c**) and the mean value for granzyme B (**b**) and TNF-α (**d**). Statistically significant differences were only shown for rLmlRAB or rLmlRABC, and were performed in each group and between groups (*P* ≤ 0.01)

We analysed IFN-γ, granzyme B, TNF-α and IL-10 levels in PBMC from individuals cured of CL (CCLm), or with a probable asymptomatic *L. major* (HHR*Lm*) or *L. infantum* (HHR*Li*) infection as well as in naïve control subjects (HLR), following stimulation with rLmlRAB or rLmlRABC (Fig. 3). Results were compared to those observed after PHA (data not shown) or SLA stimulation. It should be noted that asymptomatic infection was strongly suggested by high IFN-γ levels in response to SLA in individuals living in endemic areas for *Leishmania* infection (> 300 pg/ml).

IFN-γ levels were significantly higher in response to SLA in CCLm (Mean ± SD: 6,124.39 ± 5,363.13 pg/ml), HHR*Lm* (12,351.75 ± 9,364.14 pg/ml) and HHR*Li* (23,106.75 ± 8,929.51 pg/ml) when compared to HLR group (52.03 ± 84.99 pg/ml) (*χ* ^2^ = 31.875, *df* = 1, *P* = 0.0001; *χ* ^2^ = 25.500, *df* = 1, *P* = 0.0001; and *χ* ^2^ = 30.136, *df* = 1, *P* = 0.0001, respecively) (Fig. 3a). We showed that the full-length rLmlRAB protein was able to induce high significant levels of IFN-γ, in CCLm and HHR*Li* groups (3,720.65 ± 3,502.28 and 6,252.08 ± 3,984.55 pg/ml, respectively) (*Z* = 4.597, *P* ≤ 0.0001; *Z* = 4.457, *P* ≤ 0.0001, respectively, when compared to unstimulated cultures). This rLmlRAB-induced IFN-γ response was significantly higher than that observed in HLR group (1,098.33 ± 1,105.13 pg/ml; *χ* ^2^ = 19.7, *df* = 1, *P* = 0.0001) (Fig. 3a). Interestingly, the divergent C terminal part rLmlRABC were able to induce high significant levels of IFN-γ in all immune groups: CCLm (5,540.05 ± 4,984.83 pg/ml), HHR*Lm* (6,858.70 ± 6,084.36 pg/ml) and HHR*Li* (5911.01 ± 4859.83 pg/ml) groups (*Z* = 4.782, *P* ≤ 0.0001; *Z* = 3.549, *P* ≤ 0.0001; *Z* = 4.286, *P* ≤ 0.0001, respectively). This IFN-γ response was also specific since it was significantly higher in comparison with naïve controls (209.9 ± 216.81 pg/ml; *χ* ^2^ = 21.824, *df* = 1, *P* = 0.0001; *χ* ^2^ = 12.942, *df* = 1, *P* = 0.0003; *χ* ^2^ = 13.297, *df* = 1, *P* = 0.0001, respectively) (Fig. 3a). The full protein seems to be more immunogenic in individuals immune to *L. infantum* infection, since LmlRAB induced significantly higher IFN-γ levels in *L. infantum* compared to *L. major* immune groups. It should be noted that IFN-γ levels were significantly higher in response to rLmRABC when compared to rLmlRAB stimulation in individuals immune to *L. major* infection. Significant levels of granzyme B were detected in response to SLA stimulation in CCLm (1,345.36 ± 2,015.07 pg/ml) and HHR*Lm* (1,766 ± 1,698 pg/ml) (*Z* = 2.521, *P* = 0.011; *Z* = 2.521, *P* = 0.011, respectively) but not in HLR group (Fig. 3b). The rLmlRAB was able to induce significant granzyme B response in HHR*Lm* (976.33 ± 1,341 pg/ml) while inducing lower but significant production in HLR group (407.34 ± 245.71 pg/ml) (*Z* = 2.380, *P* = 0.017; *Z* = 2.023, *P* = 0.043, respectively). A high granzyme B response, although not significant when compared to unstimulated cultures, was observed in CCLm group (1,345.36 ± 2,015 pg/ml). This result could be due to the great variability observed in granzyme B levels in CCLm individuals together with the relative low number of samples. This cytokine was not significantly detected in response to rLmlRABC stimulation. Granzyme B response could not be analysed in HHR*Li* group. As expected, no IL-10 was observed in response to SLA. The full-length rLmlRAB as well as its divergent part rLmlRABC induced high significant levels of IL-10 in immune as well as in naïve groups, indicating that IL-10 production was not a feature of *Leishmania* stimulation (Fig. 3c). Interestingly, IFN-γ/IL-10 ratio were 6.8; 2.7 and 7 for rLmlRAB and 14.8; 7.7 and 6.2 for rLmlRABC in CCLm, HHR*Lm* and HHR*Li*, respectively. These data suggest that IL-10 inducing-capacity of rLmlRAB and rLmlRABC did not alter the ability of both proteins to induce high IFN-γ levels. TNF-α was not observed at significant levels after SLA or rLmlRAB/rLmlRABC stimulation (Fig. 3d).

### rLmlRAB and rLmlRABC induce specific IFN-γ-producing CD4+ and CD8+ T cells in individuals cured of *L. major* infection

**Fig. 4:**
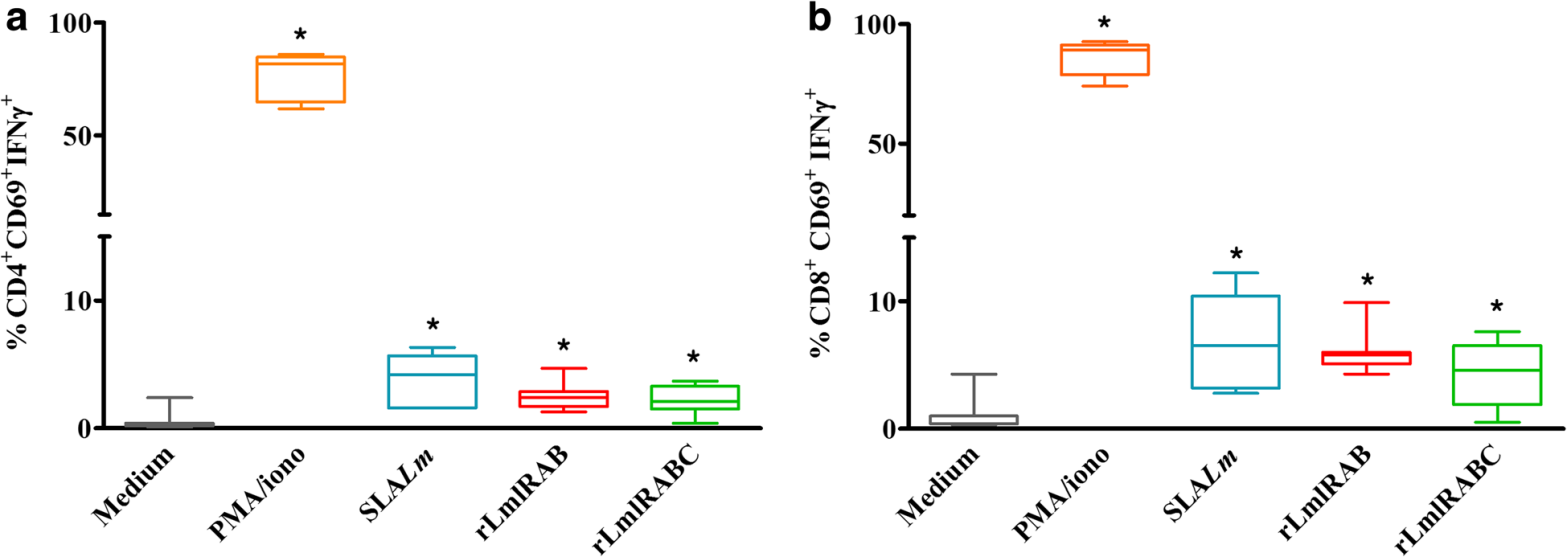
Phenotype of IFN-γ producing cells. PBMC were stimulated with PMA (50 ng/ml)/ionomicyn (10–6 M) for 6 h (positive controls), SLA (10 μg/ml), rLmlRAB or rLmlRABC (10 μg/ml) for 120 h. For intracellular IFN-γ detection, cells were treated with Golgistop for the last 6 h of culture, fixed and permeabilized using BD Cytoperm/cytofix kit. Data were analyzed by BD FACS Canto II. Results represent the frequency of IFN-γ producing cells among the CD3 + CD4+ (**a**) and CD3 + CD8+ (**b**) cell populations. Wilcoxon signed-rank test was used to compare percentage of cells producing INF-γ (horizontal bars inside the box indicate median values). *Statistically significant differences from stimulated and non-stimulated cultures (*P* < 0.05)

Phenotypic analysis of T cells producing IFN-γ in response to rLmlRAB and rLmlRABC was performed in CCLm group (*n* = 8) (Fig. 4). Results were compared to SLA-specific IFN-γ producing T cell responses. SLA induced a significant increase in the percentage of IFN-γ-producing CD4+ T cells (mean ± SD: 3.72 ± 2.06%) as well as IFN-γ-producing CD8+ T cells (6.87 ± 3.73%) among total T CD4+ cells (62.95 ± 5.94%, data not shown) and total T CD8+ cells (46.78 ± 4.29%, data not shown), respectively (*P* = 0.018). Interestingly, we showed a significantly higher percentage of CD4+ T cells producing IFN-γ (2.52 ± 1.12%) among total CD4+ T cells (59.85 ± 3.70%), after rLmlRAB stimulation in CCLm individuals, in comparison to non-stimulated cultures (*Z* = -2.366, *P* = 0.018) (Fig. 4a). A significant increase was also observed in the percentage of IFN-γ-producing CD8+ T cells (6.02 ± 1.80%) among total CD8 + T cells (49.91 ± 4.43%) (*Z* = -2.366, *P* = 0,018) (Fig. 4b). Interestingly, the divergent rLmlRABC was also able to induce a significant increase in the percentage of both IFN-γ-producing CD4+ T cells (2.28 ± 1.19%) and IFN-γ-producing CD8+ T cells (4.42 ± 2.49%) among total CD4 + T cells (59.3 ± 3.56%) and total CD8+ T cells (52.51 ± 1.9%), respectively (*Z* = -2.366, *P* = 0.018), in CCLm individuals (Fig. 4).

## Discussion

Advances in our understanding of *Leishmania* infection pathogenesis and the generation of host protective immunity, together with completed *Leishmania* genome sequences, has opened new avenues for vaccine research. To date, only one recombinant polyprotein *Leishmania* vaccine candidate, LEISH-F1, has entered phase II clinical human testing [20] and the need for the identification of new vaccine candidate molecules is crucial. We previously described a *Leishmania* specific gene encoding a large 610 amino acid RAB GTPase (LmlRAB) that is over-expressed in parasites infecting human or mouse macrophages [13, 21, 22]. It is encoded by a single copy Lmlrab gene highly conserved between *Leishmania* species, while displaying a relative low level of homology with mammalian homologues. This protein is characterized by a carboxy-terminal part extension only found in the genus *Leishmania*, suggesting that LmlRAB is a *Leishmania*-specific protein [13]. A role in phagosome maturation, regulation of the secretory pathway, regulation of both uptake and degradation of endocytosed hemoglobin, was described for RAB proteins in *Leishmania* parasites [16–18]. The present study aimed to evaluate the immunogenicity of the recombinant LmlRAB protein as well as its divergent carboxy terminal part rLmlRABC, in individuals who have developed a protective immunity against *L. major* or *L. infantum* infection. We showed that both proteins were able to induce high significant levels of IFN-γ, in individuals with a probable asymptomatic *L. major* or *L. infantum* infection or recovered from CL. It is well established that IFN-γ is a key effector cytokine crucial to eliminate *Leishmania* parasites [23]. IFN-γ levels were significantly higher in response to rLmlRABC when compared to rLmlRAB stimulation in individuals immune to *L. major* infection, suggesting that more T cell epitopes could be located in the most divergent region of the protein. Similar IFN-γ inducing capacity was observed for both proteins in *L. infantum* immune individuals. Surprisingly, the full protein induced significantly higher IFN-γ levels in *L. infantum* compared to *L. major* immune groups, suggesting that rLmlRAB could be more immunogenic in individuals immune to *L. infantum* infection. Various factors can influence immunogenicity of a protein such as differences in amino acid sequences, immunogenic dominant epitopes, individual-related factors including immune status and genetic features. Similarly, to these results, we have recently demonstrated that LaPSA-38S, a *L. amazonensis* promastigote surface antigen, induced high specific IFN-γ levels in individuals immune to *L. major* and *L. infantum* infection [24]. Our previous data and those of other authors have shown *Leishmania* protein-specific IFN-γ levels, in immune individuals [25–27]. We further showed in this study, a significant increase in the percentage of rLmlRAB and rLmlRABC-specific IFN-γ-producing CD4+ as well as CD8+ T cells, in CL recovered individuals. Several studies have shown that specific CD4+ T cells induced by total *Leishmania* antigen stimulation were the main source of IFN-γ [10, 11, 28–30]. However, few studies have analysed phenotypes of cytokine-producing T cells in response to defined *Leishmania* antigens in humans. We previously showed that LaPSA-38S stimulation was associated with an increase in CD4+ T cells producing IFN-γ□in individuals recovered from CL [24]. Other *Leishmania* proteins were described to induce CD4+ T cells producing IFN-γ in *Leishmania* asymptomatic individuals [31, 32]. CD8+ T cells were also involved in protection during human *Leishmania* infection [31–34] and were more recently described as a source of IFN-γ production in response to total antigens [9, 35, 36]. However, studies reporting the contribution of *Leishmania-*specific CD8+ T cells in IFN-γ production following defined antigens stimulation are scarce [37, 38]. The capacity of rLmlRAB and rLmlRABC to induce granzyme B production was also assessed in this work. Granzyme B is a cytolytic protein expressed by memory CD8+ and CD4+ T cells [39], which has been associated with a good prognosis in human *L. major* and *L. mexicana* CL patients [40, 41]. More recently, high levels of granzyme B as well as activated CD8+ T cells were observed in response to SLA in healed VL or PKDL individuals, indicating a possible role of CD8+ T cells in resistance to infection, *via* the perforin-granzyme B pathway [12, 42]. Furthermore, granzyme B-mediated elimination of intracellular protozoan parasites including *Leishmania,* was reported [43]. We showed that rLmlRAB protein was able to induce significant granzyme B levels in individuals with immunity to *L. major* infection. However, low but significant granzyme B levels were also detected in naïve group. Given the small sample sizes and the great variability observed in individual responses, these results need to be confirmed in larger groups and does not support the fact that this granzyme B production is not a feature of *Leishmania* infection. We recently showed a specific and significant production of granzyme B in response to LaPSA-38S protein in individuals with immunity to *L. major* [24]. Excreted/secreted *Leishmania* antigens or peptides were also described to induce granzyme B in healed CL individuals. With regard to the anti-inflammatory IL-10 cytokine, we showed that rLmlRAB and rLmlRABC induced significant levels of this cytokine in immune individuals as well as in healthy subjects. IL-10 is produced by different cell populations including macrophages, Th2 and different regulatory CD4+ T subsets and has been demonstrated to have regulatory effects on immune responses and pathology [44]. Although IL-10 overproduction has been associated to parasite persistence and disease establishment [45, 46], this cytokine may also play a role in controlling the excessive inflammatory response, when produced by inducible Treg cells (CD4 + CD25-FoxP3-), during acute infection [47]. rLmlRAB and rLmlRABC-induced IL-10 observed in immune as well as naïve individuals suggest that both proteins may contain cross-reactive epitopes that can be recognized by T cells of the majority of individuals and that this response was not a feature of *Leishmania* infection. IL-10 induction in PBMC from both immune and healthy individuals has already been described for other *Leishmania* recombinant proteins [24–26, 48, 49]. It should be noted that the presence of IL-10 did not prevent a strong IFN-γ-induced response to rLmlRAB proteins and high IFN-γ/IL-10 ratios were observed, suggesting that the IL-10 inducing capacity of both proteins does not exclude them as potential candidate vaccines. It has been shown that high ratio of IFN-γ to IL-10 provided the best correlate of protective immunity and it has been suggested that a correct balance of pro-inflammatory to regulatory cytokines may be involved in the outcome of human leishmaniasis and in the prediction of vaccine success [50, 51].

## Conclusion

In summary, we demonstrated that rLmlRAB and rLmlRABC are able to induce a predominant Th1 response in individuals immune to *L. major* or *L. infantum*, making this protein a potential cross-species vaccine candidate. As far as we know, only one study has reported the immunogenicity of a RAB antigen in a parasitic disease [52]. Our results deserve further investigations to evaluate these vaccine candidates in combination with other promising immunogenic antigens such as salivary gland proteins. Moreover, since synthetic peptide-based vaccines are the potential future of vaccination, identification of such vaccines candidates from LmlRAB proteins are under progress in our laboratory.

## Abbreviations

CBA: Cytometric bead array
CD4: Cluster of differentiation 4
CD69: Cluster of differentiation 69
CD8: Cluster of differentiation 8
CpG-ODN: CpG oligodeoxynucleotides
ELISA: Enzyme-linked immunosorbent assay
GTP: Guanosine-5′-triphosphate
IFN-γ: Interferon-gamma
IL-10: Interleukin-10
IL-13: Interleukin-13
IL-2: Interleukin-2
IL-4: Interleukin-4
IL-5: Interleukin-5
NO: Nitric oxide
PBMC: Peripheral blood mononuclear cells
PMA: Phorbo 12-Myristate 13-Acetate
RAB: Ras-related proteins in brain
RPMI-1640: Roswell Park Memorial Institute 1640 medium
SLA: Soluble *Leishmania* antigen
TGFβ: Transforming growth factor beta
Th1: Type 1 T helper lymphocytes
Th2: Type 2 T helper lymphocytes
TNFα: Tumor Necrosis Factor-alpha
